# Transcriptomic and Network Analyses Reveal Immune Modulation by Endocannabinoids in Approach/Avoidance Traits

**DOI:** 10.3390/ijms23052538

**Published:** 2022-02-25

**Authors:** Andrea Termine, Carlo Fabrizio, Juliette Gimenez, Anna Panuccio, Francesca Balsamo, Noemi Passarello, Silvia Caioli, Luana Saba, Marco De Bardi, Francesco Della Valle, Valerio Orlando, Laura Petrosini, Daniela Laricchiuta

**Affiliations:** 1 Data Science Unit, IRCCS Fondazione Santa Lucia, 00179 Rome, Italy; andreatermine544@gmail.com (A.T.); carlo.fabrizio217@gmail.com (C.F.); 2 Epigenetics and Genome Reprogramming Laboratory, IRCCS Fondazione Santa Lucia, 00179 Rome, Italy; j.gimenez@hsantalucia.it; 3 Experimental and Behavioral Neurophysiology Lab, IRCCS Fondazione Santa Lucia, 00179 Rome, Italy; anna.panuccio@gmail.com (A.P.); francesca.balsamo93@gmail.com (F.B.); noemi.passarello@gmail.com (N.P.); laura.petrosini@uniroma1.it (L.P.); 4 Department of Psychology, University Sapienza of Rome, 00185 Rome, Italy; 5 Department of Human Sciences, Guglielmo Marconi University, 00193 Rome, Italy; 6 Department of Humanities, Federico II University of Naples, 80131 Naples, Italy; 7 Unit of Neurology, IRCCS Neuromed, 86077 Pozzilli, Italy; silviacaioli@yahoo.it; 8 Department of Sciences and Technologies for Humans and Environment, University of Campus Biomedico, 00128 Rome, Italy; luana_saba@libero.it; 9 Neuroimmunology Unit, IRCCS Fondazione Santa Lucia, 00179 Rome, Italy; m.debardi@hsantalucia.it; 10 KAUST Environmental Epigenetics Program, Biological Environmental Science and Engineering Division, King Abdullah University of Science and Technology (KAUST), 4700 KAUST, Thuwal 23955, Saudi Arabia; francesco.dellavalle@kaust.edu.sa (F.D.V.); valerio.orlando@kaust.edu.sa (V.O.)

**Keywords:** transcriptomics, RNA-Seq, network analysis, endocannabinoids, endocannabinoid system, immune system

## Abstract

Approach and avoidance (A/A) tendencies are stable behavioral traits in responding to rewarding and fearful stimuli. They represent the superordinate division of emotion, and individual differences in such traits are associated with disease susceptibility. The neural circuitry underlying A/A traits is retained to be the cortico-limbic pathway including the amygdala, the central hub for the emotional processing. Furthermore, A/A-specific individual differences are associated with the activity of the endocannabinoid system (ECS) and especially of CB1 receptors whose density and functionality in amygdala differ according to A/A traits. ECS markedly interacts with the immune system (IS). However, how the interplay between ECS and IS is associated with A/A individual differences is still ill-defined. To fill this gap, here we analyzed the interaction between the gene expression of ECS and immune system (IS) in relation to individual differences. To unveil the deep architecture of ECS-IS interaction, we performed cell-specific transcriptomics analysis. Differential gene expression profiling, functional enrichment, and protein–protein interaction network analyses were performed in amygdala pyramidal neurons of mice showing different A/A behavioral tendencies. Several altered pro-inflammatory pathways were identified as associated with individual differences in A/A traits, indicating the chronic activation of the adaptive immune response sustained by the interplay between endocannabinoids and the IS. Furthermore, results showed that the interaction between the two systems modulates synaptic plasticity and neuronal metabolism in individual difference-specific manner. Deepening our knowledge about ECS/IS interaction may provide useful targets for treatment and prevention of psychopathology associated with A/A traits.

## 1. Introduction

Responding to rewarding and fearful stimuli is characterized by individual differences ranging from approach to avoidance (A/A) tendencies, behavioral traits relatively stable over time and across conditions that require balancing potential risks against rewards in uncertain environments [1,2]. In murine experimental models, when choosing between emotional conflicting drives is needed, three categories of mice can be identified (balancing (BA), avoiding (AV), and approaching (AP)) according to their A/A behavioral tendency [3,4,5,6]. At a functional level, the A/A behaviors toward novel, rewarding, and dangerous stimuli require the perception of, interaction with, and recognition of the somatosensory stimuli, as well as motivational and attentional drive, emotional response, formation of memories [7]. The A/A behaviors are influenced by multiple factors like strain, sex, age and, at structural level, they are regulated by the interaction among environmental, genetic/epigenetic, and neuronal modulation determiners [8,9,10,11,12]. The neural circuitry of the A/A behaviors has often been attributed to cortico-limbic pathways including the prefrontal cortex, amygdala, hypothalamus, and periaqueductal gray matter [13]. Both in humans and animals, among neuromodulatory factors the endocannabinoid system (ECS) plays a noticeable role in regulating the A/A behaviors [4,14,15,16,17]. The ECS is a neuromodulatory system, which acts as a retrograde feedback mechanism at both excitatory and inhibitory synapses, primarily responsible for maintaining homeostasis, balance in internal environment (temperature, mood, and immune system), and energy input and output in biological systems [18,19]. It comprises classical (Cannabinoid receptor type 1 and 2; CB1, CB2) and non-classical (transient receptor potential cation channel subfamily V member 1 and 2; TRPV1 and TRPV2) receptors, their endogenous ligands (endocannabinoids (EC): anandamide (AEA), 2-arachidonoylglycerol (2-AG), 2-arachidonyl glyceryl ether (2-AGE), N-arachidonoyl dopamine (NADA), palmitoylethanolamide (PEA)), and enzymes involved in endocannabinoid metabolism (fatty acid amide hydrolase (FAAH), N-acyl phosphatidylethanolamine phospholipase D (NAPEPLD), palmitoylethanolamide-preferring acid amidase (PAA)) [20]. While CB2 receptors are primarily expressed in the cells of the immune system, CB1 receptors are densely present in the brain areas responsible for learning and memory, movement, hormone regulation, body temperature, sensory perception, reward, emotions, and individual differences traits [21].

Critically for the present study, the three aforementioned categories of AV, BA, and AP mice differ in CB1 receptor expression in amygdala, central limbic hub for the emotional processing. Noticeably, AV and AP mice display a higher CB1 receptor density and functionality in comparison to BA mice [3]. Within the amygdala, CB1 receptors are abundant and presynaptically present only on GABAergic interneurons, which inhibit glutamatergic pyramidal neurons [22]. Thus, CB1 receptors on GABAergic interneurons mediate retrograde signaling and depolarization-induced suppression of inhibition. In other words, in amygdala the CB1 receptor activation efficiently inhibits GABA release, controlling the efficacy of its own synaptic input in an activity-dependent manner and potentiating the disinhibition of amygdala pyramidal neurons [22]. In turn, the increased excitatory activity of amygdala pyramidal neurons could increase the output to the other cortico-limbic structures influencing the processing of emotional stimuli and attributing major salience to them, regardless of if they are pleasant or aversive. Thus, the described CB1-mediated amygdaloid mechanism sustains the A/A responses. In fact, the link between ECS and A/A traits has been confirmed by human studies in which variants in ECS genes have been associated with several A/A personality traits, including neuroticism and agreeableness [23]. Accordingly, CB1 signaling-A/A traits relation has been also suggested by the ECS involvement in social play of adolescent rodents, a highly rewarding behavior with clear sex-differences [24]. In juvenile rodents, an increased EC tone increases approaching behavior toward playing with a conspecific, while a block of CB1 receptors reduces it, with a sex-specific pattern related to a reduced density of astrocytes specifically in the amygdala [25]. Taken as a whole, these findings emphasize the association of ECS activity, and especially CB1 receptors, with individual differences in A/A traits, and demonstrate regional and circuit-specific effects of EC signaling on emotional processes.

Furthermore, it has been shown that individual differences, such as behavioral phenotypes or human personalities, are associated with disease susceptibility through immune regulation, even if the mechanisms do not converge on a clear pattern [26,27,28]. It has been reported that human individuals characterized by avoiding traits are prone to developing conditions that are broadly considered to reflect a T-helper 2 cell immune polarization [29,30,31], and to show elevated basal glucocorticoid production [32,33], associated with health and immune negative outcomes [34,35,36,37]. Accordingly, rodents characterized by avoiding traits, defined as having consistently slower-than-median approaching latencies in two novel conditions, have low-grade elevation in glucocorticoid production which is associated with shortened life span [9,10,38]. Furthermore, Michael et al. (2020) suggested that avoiding traits are associated with a glucocorticoid resistant state with poorly regulated innate inflammation and dampened cell-mediated immune responses, probably associated with exaggerated T-helper 2 responses [39]. 

Remarkably, ECS signaling contributes to maintaining the immune homeostasis, limiting the spontaneous activation of immune cell function [40,41,42,43,44]. Multiple studies have linked ECS activation with inflammatory processes, by mediating mature immune cell trafficking, fostering cytokine production, leukocyte function, and dendritic cell recruitment during the immune response [45,46].

To date, information on the relationship among A/A traits, ECS, and immune function is lacking. To fill this gap, in the present study the interaction between the gene expression of ECS and immune system (IS) was analyzed in relation to individual differences. To unveil the deep architecture of ECS-IS interaction, we used a cutting-edge tool: we performed cell-specific high-throughput stranded RNA-sequencing of amygdala pyramidal neuron from AV, BA, and AP mice and applied deep transcriptomic analyses consisting in differential gene expression profiling, functional enrichment, and protein–protein interaction network analyses. Results showed that interplay between the two systems is able to modulate synaptic plasticity and neuronal metabolism in an individual-difference-specific manner.

## 2. Results

### 2.1. Altered Immune System Activity in the Pyramidal Neurons of Mice Characterized by A/A Traits

In Thy-1 YFP pyramidal neurons of amygdala, differential expression analysis identified 5480 significant DEGs in the comparison between BA and AV mice (2630 up-regulated; 2850 down-regulated), and 886 significant DEGs between BA and AP mice (531 up-regulated; 355 down-regulated).

**Figure 1:**
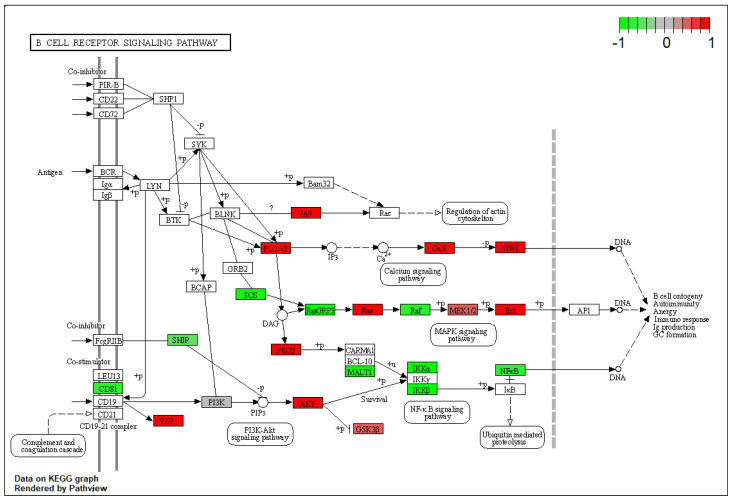
Kyoto Encyclopedia of Genes and Genomes B Cell receptor signaling pathway rendered by pathview. Colored boxes represent differentially expressed genes found in this pathway. The colorbar indicates scaled log2-fold-change values in gene expression of amygdala pyramidal neurons of AV over BA mice.

In the comparison between BA and AV mice, several DEGs belonging to the pro-inflammatory cytokines including tumor necrosis factor α (TNFα), interleukin-2 (IL-2), and interleukin-12 (IL-12), were identified (Supplementary Table S1). Indeed, the expression of genes involved in the production and regulation of cytokines significantly differed between BA and AV mice, since DEGs significantly enriched IL-2 and IL-12 production pathways (GO:0032623; p._adjusted_ = 0.03; GO:0032615; p._adjusted_ = 0.03), as well as the T-cell receptor signaling pathway (KEGG:mmu04660; p._adjusted_ = 0.03), and the inflammatory mediator regulation of TRP channels pathway (KEGG:mmu04750, p._adjusted_ = 0.007) (Supplementary Table S1). Such an alteration of immune response between phenotypes involved DEGs belonging to the chemokine (KEGG:mmu04062; p._adjusted_ < 0.001) and RAS signaling (KEGG:mmu04014, p._adjusted_ < 0.001) pathways, resulting in differential modulation of the B-cell receptor signaling (p._adjusted_ < 0.001) pathway (Figure 1). 

In the comparison between BA and AP mice, the AP mice showed a clear inflammatory pattern with the differentially expressed B-cell receptor (KEGG: mmu04662; p._adjusted_ < 0.01) and the chemokine signaling (KEGG:mmu04062; p._adjusted_ < 0.01) pathways.

Therefore, we identified an alteration in the expression of immune-related genes in the amygdala pyramidal neurons of mice characterized by A/A traits, in comparison to BA mice.

### 2.2. Altered ECS Activity in the Pyramidal Neurons of Mice Characterized by A/A Traits

**Figure 2:**
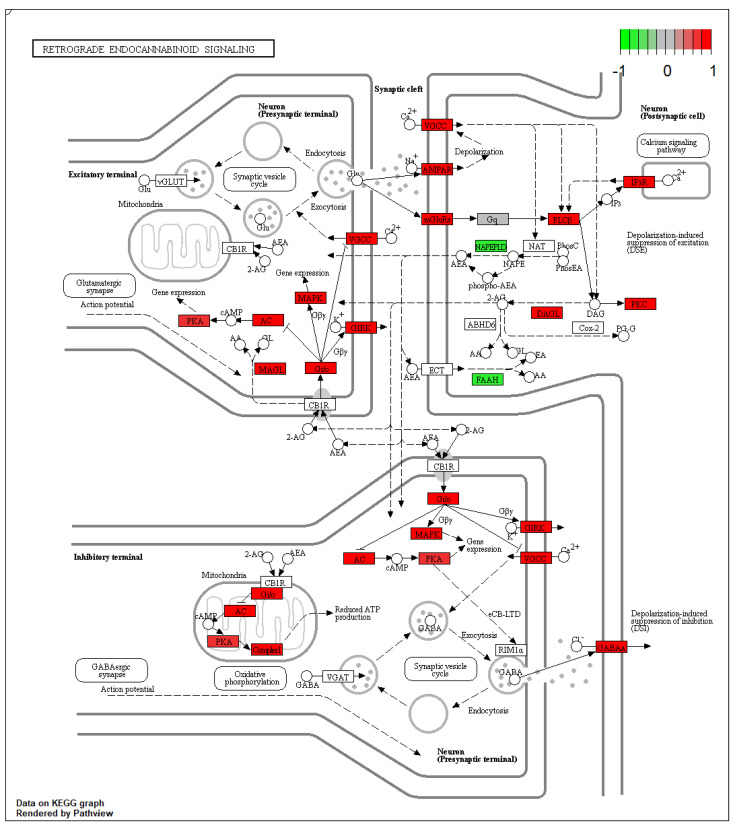
Retrograde endocannabinoid signaling pathways show the alteration of oxidative phosphorylation in mitochondria coupled with alterations in both excitatory (glutamatergic) and inhibitory (GABAergic) synaptic terminals.

**Figure 3:**
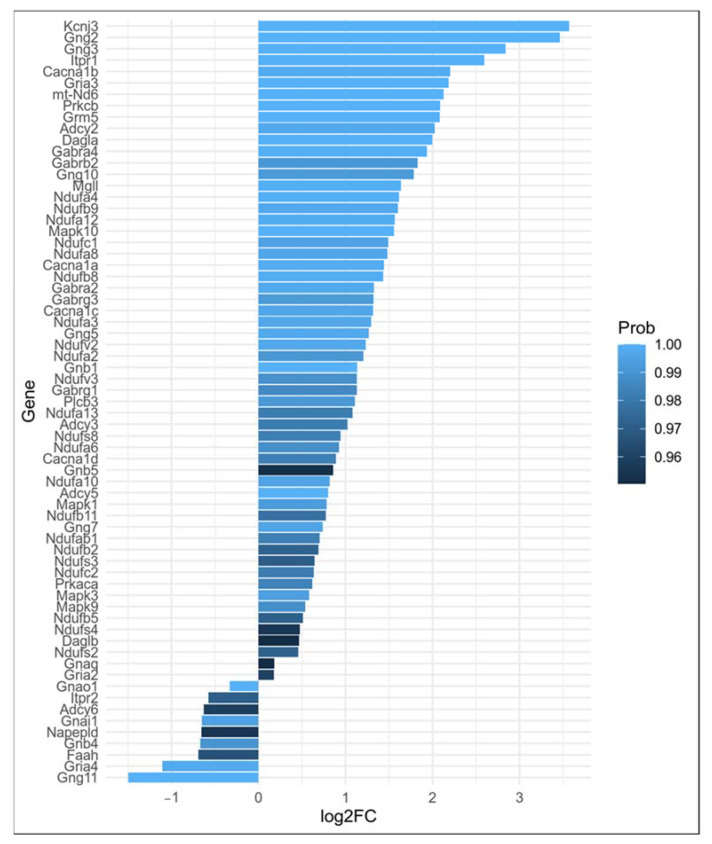
Barplot representing differential expression of genes belonging to retrograde ECS signaling pathway along with genes from pro-inflammatory IL-2 and IL-12 production pathways seen in Section 2.1. These genes will be used in Section 2.3 to study the interplay between ECS and IS.

In Thy-1 YFP pyramidal neurons of amygdala from AV mice, 68 DEGs belonging to the retrograde ECS signaling pathway (KEGG:mmu04723; p._adjusted_ < 0.001) were identified in comparison to BA mice (Figure 2). Notably, *FAAH* and *NAPEPLD* genes were significantly down-regulated and the *DAAG* genes were over-expressed in AV mice when compared to BA mice (Figure 3).

In Thy-1 YFP pyramidal neurons of amygdala from AP mice compared to BA mice, the obtained DEGs did not enrich any classical ECS pathway, as CB1 and CB2. However, we found that inflammatory mediator regulation of TRP channels was significantly altered (KEGG:mmu04750; p._adjusted_ < 0.05). 

### 2.3. The Immune and ECS Interplay in the Pyramidal Neurons of Mice Characterized by the Avoiding Trait

Having identified significant alterations of both immune- and ECS-related genes in the comparison between AV and BA mice, we further investigated if these systems were interconnected. Thus, a PPI network was used to investigate the interplay between DEGs of ECS and IS, obtaining a significant PPI score (*p* < 0.0001).

**Figure 4:**
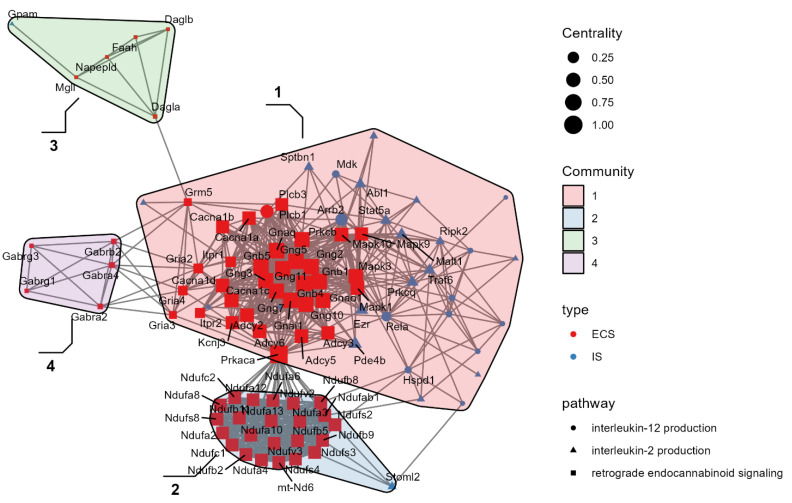
Network analysis. Differentially expressed genes (DEGs) from retrograde endocannabinoid signaling are represented in red, while DEGs from IL-2 and IL-12 production systems are represented in blue. Four communities have been identified: Community 1: glutamatergic synapse; *p* < 0.00001; Community 2: mitochondrial respiratory chain complex I; *p* < 0.00001. Community 3: anandamide metabolism; *p* < 0.00001. Community 4: GABAergic transmission GABA receptor complex; *p* < 0.00001.

Based on the network analysis, we identified four communities of functionally connected genes, demonstrating the interaction between DEGs of ECS and pro-inflammatory IL-2 and IL-12 production pathways in communities 1 and 2 (Figure 4).

**Figure 5:**
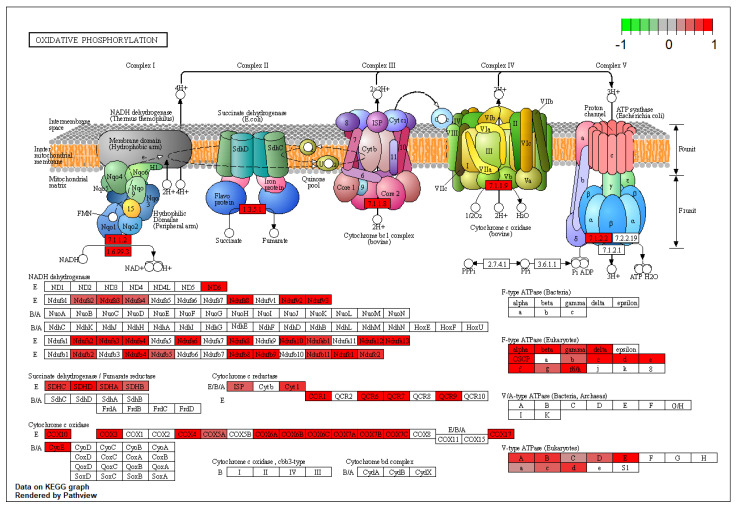
Oxidative phosphorylation pathway. Data show a substantial alteration of NADH components on the inner side of the mitochondrial membrane driven by COX and Ndufs-family genes.

In particular, community 1 enriched the glutamatergic synapse pathway and included all DEGs of IL-2 and IL-12 production systems (*p* < 0.00001) and of ECS with MAPK1, MAPK3, and MAPK10, representing the connection hubs between the IS and ECS components (Community 3: anandamide metabolism; *p* < 0.0001) in the glutamatergic and GABAergic transmission (Community 4: GABA receptor complex; *p* < 0.0001) (Figure 3). Furthermore, community 2 enriched the mitochondrial respiratory chain complex I (KEGG:mmu00190; *p* < 0.0001). Specifically, community 2 showed a dense overexpression pattern of cytochrome c oxidase DEGs that regulate the inflammation in association with several genes coding for NADH subunits (Figure 5).

**Figure 6:**
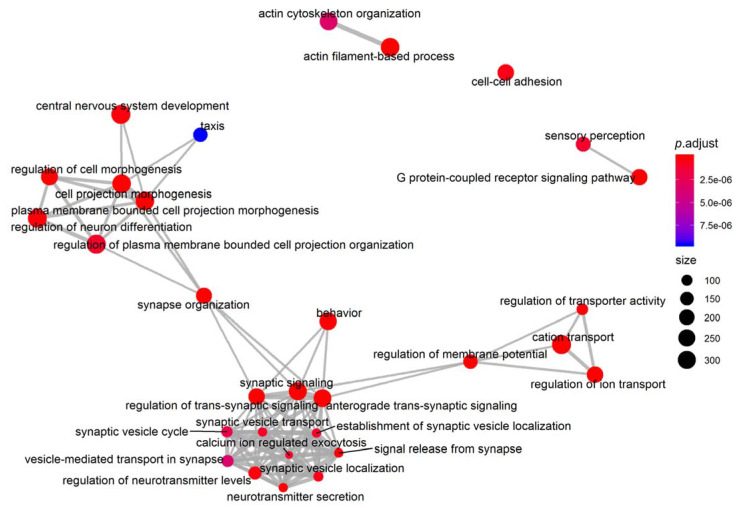
Network analysis and enrichmap on Biological Process, a component of Gene Ontology analysis. Data show an overall modulation of synapse plasticity and neurotransmission.

In the comparison between BA and AV phenotypes, the neurotransmission and synaptic plasticity were the most altered functions. In fact, another network analysis on GO terms in the Biological Process component of Gene Ontology showed three functional modules linking together neurotransmission regulation (GO:0099177, regulation of trans-synaptic signaling; *p* < 0.0001), electrophysiological activity (GO:0043269, regulation of ion transport; *p* < 0.0001), and neuronal plasticity (GO:0048858, cell projection morphogenesis; *p* < 0.0001). These three modules converge on the synapse organization (GO:0050808; *p* < 0.0001) and regulation of membrane potential (GO:0042391; *p* < 0.0001) pathways, resulting thus in a modulation of behavior (GO:0007610; *p* < 0.0001) (Figure 6).

## 3. Discussion

Individual differences in A/A traits originate from differences in predisposition toward rewarding or punitive stimuli, respectively. While most studies addressed the differences in the conflict paradigms by manipulating the amygdala-frontal network [4,5,7,47,48,49,50,51,52], in the present study we faced the A/A traits investigating the molecular differences. Interestingly, the spontaneous individual differences that characterize the three sub-populations of AV, BA, and AP mice are associated with differences in CB1 density in the amygdala [3,4,5]. As described by the extensive literature on the ECS modulation of IS, the EC receptors are expressed also on immune cells [53,54]. Furthermore, molecules previously thought to be specific for the immune-cells, such as pro- and anti-inflammatory cytokines, can be also produced and released from neurons. 

In the present research, to investigate the interaction between ECS and IS, we performed both RNA-Seq on the pyramidal neurons of the amygdala and bioinformatics analyses in the AV, BA, AP phenotypes. 

We identified several altered pro-inflammatory pathways in the pyramidal neurons of the amygdala of AV when compared to the BA mice. In particular, we found in AV mice the alteration of the pathways leading to the production of TNFα, IL-2, and IL-12. The production of these pro-inflammatory cytokines is tightly regulated by the superfamily of transient receptor potential (TRP) and by the B-cell receptor signaling pathways controlling cell differentiation and cytotoxicity. Further, TRP action is facilitated by pro-inflammatory chemokines, responsible for the recruitment of immune cells [55,56,57]. Such findings indicate the chronic activation of adaptive immune response [58]. 

Although we did not identify any direct difference in the activation of the ECS in AP mice, in comparison to BA mice, the same pattern of inflammation was found in AP and AV mice when compared to BA mice. In fact, the B-cell receptor and chemokine signaling pathways as well as modulation of the inflammatory response by TRP channels were altered in both AP and AV mice. This evidence suggests that the opposite approach and avoidance behaviors may be related to a unique molecular background. In other words, we are advancing that the up- or down-regulation of the genes involved in the interplay between ECS and IS may be a building block of the switch between approach and avoidance. 

Since EC interact with and are expressed by almost all immune cells [46], ECS contributes to maintaining the immune homeostasis, functioning as a gate-keeper of IS [40,41,42,43,44] through the activation of the CB1 and CB2 receptors as well as of TRPV1 and TRPV2 receptors. The TRP receptors, mainly TRPV1, are EC targets implied in the functionality of CB1 and in EC degradation [57,59,60]. We found that AV mice displayed an altered retrograde ECS signaling, with NAPEPLD and FAAH coding genes significantly downregulated. It has been proposed that the inhibition of FAAH increases CB1 activity [18]. However, FAAH inhibition also results in increased activation of other receptors, such as TRPV1 and peroxisome proliferator-activated receptor-α (PPARα) [18,61]. These non-classical receptors often have roles opposite to classical EC receptors, hindering the anti-inflammatory role of CB1 in immune modulation [53]. Thus, the downregulation of FAAH expression might sustain inflammatory processes in AV mice. Subsequently, we applied network analysis to further analyze the pattern of co-expression of IS and ECS genes in the regulation of avoidance behavior. Interestingly, in AV mice, ECS and IS genes were mostly co-expressed in the altered glutamatergic synapse pathway of the amygdala, in which the activity-dependent flow of glutamate and EC controls the secretion of pro-inflammatory cytokines through the activity of CB1 and TRP receptors in general [62], and likely of TRPV1 in particular. 

This study was performed with limited sample size, as the study of individual differences needs highly curated and homogeneous, and thus inevitably restricted groups. When coming to high-throughput sequencing for gene expression data, a small sample size results in HDLSS (high dimension, low sample size) data problems. This hindered possibilities with the network analysis, forcing us to study gene interactions by using STRINGdb. Although our study was limited to bioinformatics analyses of gene expression from RNA-Seq data, and no functional experiments were conducted, we were able to study gene networks and to map genes to known biological pathways, which contributed to the understanding of ECS/IS interaction. 

To further investigate the role of such an interaction in conflict behaviors, the chemical blockade of enzymes linked to ECS metabolism (such as FAAH or NAPEPLD) could be performed. Moreover, anti-inflammatory drugs could be administered to investigate whether AV or AP phenotypes shift toward a balanced approach to conflicting stimuli. Given that the A/A traits represent psychological risk factors [63], shedding light on ECS/IS interaction in such behaviors may provide useful targets for their treatment and prevention.

## 4. Materials and Methods

### 4.1. Subjects and Experimental Procedures

B6.Cg-Tg(Thy1-YFP)HJrs/J (Thy1-YFP; Jackson Laboratories, Bar Harbor, Maine, USA) transgenic mice were used in the present study. Thy1-YFP mice express yellow fluorescent protein (YFP) transgene driven by the thymus cell antigen 1 (Thy1) promoter that targets the pyramidal neurons of the neocortex (layer 5), amygdala, and hippocampus, and to a lesser extent the cerebellar mossy fibers, neurons in the thalamus, midbrain and brainstem, and olfactory bulb mitral cells. Transgene-expressing neurons are morphologically and physiologically comparable to non-mutant neurons.

The animals were group-housed (4 mice/cage) with standard food (Mucedola, Milan, Italy) and water ad libitum, and kept under a 12-h light/dark cycle (with the light on at 07:00 h), controlled temperature (22–23 °C), and constant humidity (60 ± 5%). The behavioral testing took place during the light phase. All efforts were made to minimize animal suffering and to reduce their number, in accordance with the European Directive (Directive 2010/63/EU). The animals assigned to the same experimental group were never siblings.

The first step of the present research was the categorization of mice according to their tendency to A/A responses. To this aim, a sample of transgenic mice (Thy1-YFP; N = 50) was submitted to the A/A Conflict Task. By means of this task, we selected transgenic mice which spontaneously responded with balanced behavior (BA; N = 3), and transgenic mice which spontaneously responded with avoiding (AV; N = 3) or approaching (AP; N = 3) behaviors in the presence of the same conflicting stimuli [3,4]. One month after the end of behavioral task, the AV, BA, and AP mice were sacrificed to sort the amygdala Thy1-YFP pyramidal neurons in order to perform the transcriptomic cell-specific RNA-analyses, tool for unveiling the deep architecture of ECS-IS interaction. To verify whether and which transcriptomic elements (especially linked to endocannabinoid system and immune system) were associated with the specific and stable predisposition to A/A behavior, the transcriptomic analyses were evaluated in the three phenotypes at a time-point distant from any behavioral testing, to rule out any acute effect of the behavioral performance on gene expression levels.

### 4.2. A/A Conflict Task

As previously described [3,5], the apparatus consisted of a Plexiglas Y-maze with a starting gray arm from which two arms (8 cm wide, 30 cm long, and 15 cm high) stemmed, arranged at an angle of 90 ° to each other. A T-guillotine door was placed at the end of the starting arm to prevent backward movements of the animal. An arm entry was defined as four legs entering one of the arms. The two choice arms differed in both color and brightness. One of the two arms had a black and opaque floor and walls, and no light inside, while the other had a white floor and walls, and was lit by a 16-W neon lamp. Notably, the colored “furniture” and the neon lamp were exchangeable between arms to alternate the spatial positions of the white and black arms. The apparatus was placed in a room that was slightly lit by a red light (40 W). It was always thoroughly cleaned with 70% ethanol and dried after each trial to remove scent cues. At the end of each choice arm, there was a blue chemically inert tube cap (3 cm in diameter, 1 cm deep) used as a food tray that prevented mice from seeing the reward at a distance but allowed easy access to the reward. Because appetites for palatable foods have to be learned, a week before testing the animals were exposed in their home cages for three days to the novel palatable food (Fonzies, KP Snack Foods, Munchen, Germany). At the end of this phase and during successive testing, to increase the motivation to search for the reward, the animals were slightly food deprived by limiting food access to 12 h/day. About 24 h after the habituation to the apparatus, the slightly food-deprived mice were submitted to two 10-trial sessions of the testing. In Session 1 (S1), the animal was placed in the starting stem and could choose to enter one of the two arms, both containing the same standard food reward. After eating, the animal was allowed to stand in its cage for a 1 min-inter-trial interval. In Session 2 (S2), which started 24 h after S1, the white arm was rewarded with the highly palatable food (Fonzies), while the black arm remained rewarded with the standard food. Notably, the A/A Conflict Task requires to choose between two conflicting drives: reaching a palatable reward placed in an aversive (white and lighted) environment, or reaching a standard food placed in a reassuring (black and dark) environment. The A/A Conflict Index represents the difference in the number of white choices between S1 and S2. Given that this index was normally distributed (mean_Δ_ = 1, SD = ±1.7), it allowed identifying the specific phenotypes of the mice [4]. In particular, in the presence of conflicting inputs, we identified 3 BA mice that reacted with balanced responses between A/A traits and their values in the A/A Conflict Index corresponded to the mean of the distribution. Furthermore, we identified 3 AV mice that exhibited avoiding responses to the conflicting stimuli and that had values of the A/A Conflict Index corresponding to minus two standard deviations of the mean. We identified also 3 AP mice that exhibited approaching responses to the conflicting stimuli and that had values of the A/A Conflict Index corresponding to minus two standard deviations of the mean. 

### 4.3. Amygdala Pyramidal Neuron-Specific RNA Sequencing

#### 4.3.1. Dissociation of Amygdala Tissue for Fluorescence-Activated Cell Sorting (FACS)

One month after the end of A/A behavioral task, the brains of AV, BA, and AP mice were cut to take bilateral amygdala 1-mm punches. Manual and enzymatic dissociations were performed using the Neural Tissue Dissociation Kit (P) (Miltenyi Biotec, Bergisch Gladbach, Germany) with some modifications. Each solution was kept on ice to minimize RNA degradation. Pipette tips were pre-coated in a 0.2 µM filtered 1× PBS-0.5% BSA solution (DPBS without Mg^2+^ and Ca^2+^, Gibco by Life Technologies, Grand Island, NY, USA; BSA Fraction V (pH 7.0), PanReac AppliChem GmbH, Darmstadt, Germany). Briefly, the amygdala punches were placed on a 35-mm diameter Petri dish, cut into small pieces using a scalpel, and 1 mL of cold Hanks’ Balanced Salt Solution without Mg^2+^ and Ca^2+^ (HBBS w/o) (Sigma-Aldrich, St. Louis, MO, USA) was added. The tissue was transferred into a 1.5 mL protein LoBind tube. Additional 1 mL HBBS w/o was used to rinse the dish and added to the 1.5 mL tube. Tissue was centrifuged at 300× *g* for 2 min at room temperature, and the supernatant was carefully aspirated. Then, 975 μL of pre-heated enzyme mix 1 (enzyme P 25 μL, buffer × 950 μL) was added to the tissue, and the 1.5 mL tube was incubated for 15 min at 37 °C under slow, continuous rotation using the MACSmix Tube Rotator (Miltenyi Biotec, Bergisch Gladbach, Germany). Then, 15 μL enzyme mix 2 (enzyme A 5 μL, buffer Y 10 μL) was added to the sample. The sample was gently inverted to mix and mechanically dissociated using the wide-tipped fire-polished Pasteur pipette by pipetting up and down 10 times slowly, followed by a further incubation in the rotator for 10 min at 37 °C under slow rotation. The second round of mechanical dissociation was performed using serially fire-polished filtered-glass Pasteur pipettes with gradual diameter diminution, and pipetting slowly up and down 10 times with each pipette, or until no tissue pieces were observable. The sample was again incubated at 37 °C for 10 min using rotator under slow rotation. The sample was strained through a MACS Smart Strainer (70 μm) (Miltenyi Biotec, Bergisch Gladbach, Germany), placed on a 15 mL tube, pre-coated with 0.2 μM filtered 1× PBS-0.5% BSA, using 8 mL of HBBS with Mg^2+^ and Ca^2+^. Cells were pelleted by centrifugation at 300× *g* for 10 min at room temperature. In order to increase the material, the supernatant was transferred into a new 15 mL tube, and centrifuged again at 300× *g* for 10 min at room temperature. The second supernatant was discarded, and the pellets obtained from these two centrifugations were pooled into a 1× PBS-0.5% BSA pre-coated SNAP-cap tube containing 1 mL of PBS. Finally, 20U Superase-Inhibitor (Ambion, Invitrogen, ThermoFisher Scientific, Walthem, MA, USA) was added and samples were stored on ice up to sorting.

#### 4.3.2. Cell Sorting and Isolation of Purified Pyramidal Neurons

For the instrument set-up, the samples collected from the amygdala of wild-type YFP-negative mice were used to gate YFP-positive neurons based on forward scatter (FSC) and side scatter (SSC) light scattering and to set YFP negativity. Afterwards, amygdala samples were collected from the Thy1-YFP AV, BA, and AP mice and stained with 1 μL of propidium iodide (PI) in order to identify dead cells. Pyramidal neurons were then sorted by using the MoFlo Astrios EQ (Beckman Coulter, Brea, CA, USA) and the pyramidal neurons characterized by YFP were collected on the basis of their physical parameters, singlets, PI negative (live cells), and YFP intensity. For initial characterization, samples were collected in PBS and samples were examined under a fluorescent microscope to verify correct sorting. Thereafter, cells were sorted directly into ice-cold lysis buffer (Reliaprep RNA Cell Miniprep System, Promega, Fitchburg, WI, USA), mixed by vortexing, kept on ice, and then stored at −80 °C until RNA extraction.

#### 4.3.3. RNA-Seq Library Preparation

After thawing on ice in presence of additional proteinase K, RNA was isolated according to manufacturer’s instructions including on-column DNase treatment. RNA samples were quantified and the quality was tested by Agilent 2100 Bioanalyzer RNA assay (Agilent Technologies, Santa Clara, CA, USA) or Caliper (PerkinElmer, Waltham, MA, USA).

Library preparation and sequencing were performed at IGATechnology (Udine, Italy). At least three independent biological replicates were used for each group. Each replicate corresponds to the amygdala of a single Thy1-YFP mouse.

Libraries were generated from each sample individually, starting from 0.06–4.41 ng of total RNA, using the Ovation SoLo RNA-Seq kit for Ultra-low input (NuGEN, Tecan Genomics, Redwood City, CA, USA), following the manufacturer’s instructions (library type: fr-second strand). Final libraries were checked with both Qubit 2.0 Fluorometer (Invitrogen by Life technologies, Carlsbad, CA, USA) and Agilent Bioanalyzer DNA assay (Agilent Technologies, Santa Clara, CA, USA) or Caliper (PerkinElmer, Waltham, MA, USA). Libraries were then prepared for sequencing and sequenced on paired-end 2 × 75 bp mode on NextSeq500 (Illumina, San Diego, CA, USA) producing 33.9 MR on average (min 29.3 MR, max 40.7 MR). For the processing of raw data (format conversion and de-multiplexing), Bcl2Fastq 2.20 version of the Illumina pipeline was used. 

Sequencing data have been deposited in the NCBI Short Read Archive (https://www.ncbi.nlm.nih.gov/geo; GEO accession number GSE196849; accessed on 19 February 2022).

### 4.4. Differential Expression Analysis

After TMM normalization and low counts filtering, the resulting genes (AMY = 9872) underwent the downstream analysis. Batch effect correction was applied with ARSyN and a principal component analysis (PCA) was performed to assess sample clustering based on their expression profiles. After PCA, 1 AV mouse was identified as outlier and removed from downstream analyses. Differentially expressed genes (DEGs) were identified using nonparametric analysis for biological replicates included in the NOISeq library. Significant differentially expressed genes were identified for a *q* > 0.95, equivalent to a *p* < 0.05 after FDR correction [64]. Subsequently, Gene Ontology (GO) and Kyoto Encyclopedia of Genes and Genomes (KEGG) Over-Representation Analyses (ORAs) were performed using Clusterprofiler to identify enriched pathways. The obtained GO terms were further clustered using GOSemSim and visualized using pathview [65] and the enrich map method to visualize and interpret results [66,67]. All biostatistical analyses were performed in R v.4.1 [68]. DEGs from retrograde endocannabinoid signaling and immune system were enriched on STRINGdb [69] to assess shared functions and known co-expression patterns, along with their protein–protein interaction (PPI) enrichment scores. 

## Data Availability

All data supporting the findings of this study are available from the corresponding author upon reasonable request.

## Supplementary Materials

The following are available online at https://www.mdpi.com/article/10.3390/ijms23052538/s1.

## References

[1] Caruso M.J., McClintock M.K., Cavigelli S.A. (2014). Temperament Moderates the Influence of Periadolescent Social Experience on Behavior and Adrenocortical Activity in Adult Male Rats. Horm. Behav..

[2] Rossier D., La Franca V., Salemi T., Natale S., Gross C.T. (2021). A Neural Circuit for Competing Approach and Defense Underlying Prey Capture. Proc. Natl. Acad. Sci. USA.

[3] Laricchiuta D., Rojo M.L., Rodriguez-Gaztelumendi A., Ferlazzo F., Petrosini L., Fowler C.J. (2012). CB_1_ Receptor Autoradiographic Characterization of the Individual Differences in Approach and Avoidance Motivation. PLoS ONE.

[4] Laricchiuta D., Rossi S., Musella A., De Chiara V., Cutuli D., Centonze D., Petrosini L. (2012). Differences in Spontaneously Avoiding or Approaching Mice Reflect Differences in CB1-Mediated Signaling of Dorsal Striatal Transmission. PLoS ONE.

[5] Laricchiuta D., Saba L., De Bartolo P., Caioli S., Zona C., Petrosini L. (2016). Maintenance of Aversive Memories Shown by Fear Extinction-Impaired Phenotypes Is Associated with Increased Activity in the Amygdaloid-Prefrontal Circuit. Sci. Rep..

[6] Laricchiuta D., Andolina D., Angelucci F., Gelfo F., Berretta E., Puglisi-Allegra S., Petrosini L. (2018). Cerebellar BDNF Promotes Exploration and Seeking for Novelty. Int. J. Neuropsychopharmacol..

[7] Elliot A.J. (2008). Handbook of Approach and Avoidance Motivation.

[8] Bell A.M., Hankison S.J., Laskowski K.L. (2009). The Repeatability of Behaviour: A Meta-Analysis. Anim. Behav..

[9] Cavigelli S.A., McClintock M.K. (2003). Fear of Novelty in Infant Rats Predicts Adult Corticosterone Dynamics and an Early Death. Proc. Natl. Acad. Sci. USA.

[10] Cavigelli S.A., Ragan C.M., Michael K.C., Kovacsics C.E., Bruscke A.P. (2009). Stable Behavioral Inhibition and Glucocorticoid Production as Predictors of Longevity. Physiol. Behav..

[11] Réale D., Reader S.M., Sol D., McDougall P.T., Dingemanse N.J. (2007). Integrating Animal Temperament within Ecology and Evolution. Biol. Rev..

[12] Roberts B.W., Walton K.E., Viechtbauer W. (2006). Patterns of Mean-Level Change in Personality Traits across the Life Course: A Meta-Analysis of Longitudinal Studies. Psychol. Bull..

[13] Laricchiuta D., Petrosini L. (2014). Individual Differences in Response to Positive and Negative Stimuli: Endocannabinoid-Based Insight on Approach and Avoidance Behaviors. Front. Syst. Neurosci..

[14] Lafenêtre P., Chaouloff F., Marsicano G. (2009). Bidirectional Regulation of Novelty-Induced Behavioral Inhibition by the Endocannabinoid System. Neuropharmacology.

[15] Pattij T., Janssen M.C., Schepers I., González-Cuevas G., De Vries T.J., Schoffelmeer A.N. (2007). Effects of the Cannabinoid CB1 Receptor Antagonist Rimonabant on Distinct Measures of Impulsive Behavior in Rats. Psychopharmacology.

[16] Van Laere K., Goffin K., Bormans G., Casteels C., Mortelmans L., de Hoon J., Grachev I., Vandenbulcke M., Pieters G. (2009). Relationship of Type 1 Cannabinoid Receptor Availability in the Human Brain to Novelty-Seeking Temperament. Arch. Gen. Psychiatry.

[17] McDonald J., Schleifer L., Richards J.B., de Wit H. (2003). Effects of THC on Behavioral Measures of Impulsivity in Humans. Neuropsychopharmacology.

[18] Cristino L., Bisogno T., Di Marzo V. (2020). Cannabinoids and the Expanded Endocannabinoid System in Neurological Disorders. Nat. Rev. Neurol..

[19] Rumińska A., Dobrzyń A. (2012). The Endocannabinoid System and Its Role in Regulation of Metabolism in Peripheral Tissues. Postepy Biochem..

[20] Zou S., Kumar U. (2018). Cannabinoid Receptors and the Endocannabinoid System: Signaling and Function in the Central Nervous System. Int. J. Mol. Sci..

[21] Pandey R., Mousawy K., Nagarkatti M., Nagarkatti P. (2009). Endocannabinoids and Immune Regulation. Pharmacol. Res..

[22] Katona I., Rancz E.A., Acsády L., Ledent C., Mackie K., Hájos N., Freund T.F. (2001). Distribution of CB1 Cannabinoid Receptors in the Amygdala and Their Role in the Control of GABAergic Transmission. J. Neurosci..

[23] Juhasz G., Chase D., Pegg E., Downey D., Toth Z.G., Stones K., Platt H., Mekli K., Payton A., Elliott R. (2009). CNR_1_ Gene Is Associated with High Neuroticism and Low Agreeableness and Interacts with Recent Negative Life Events to Predict Current Depressive Symptoms. Neuropsychopharmacology.

[24] Vanderschuren L.J.M.J., Achterberg E.J.M., Trezza V. (2016). The Neurobiology of Social Play and Its Rewarding Value in Rats. Neurosci. Biobehav. Rev..

[25] Trezza V., Damsteegt R., Manduca A., Petrosino S., Van Kerkhof L.W., Pasterkamp R.J., Zhou Y., Campolongo P., Cuomo V., Di Marzo V. (2012). Endocannabinoids in Amygdala and Nucleus Accumbens Mediate Social Play Reward in Adolescent Rats. J. Neurosci..

[26] Capitanio J.P., Abel K., Mendoza S.P., Blozis S.A., McChesney M.B., Cole S.W., Mason W.A. (2008). Personality and Serotonin Transporter Genotype Interact with Social Context to Affect Immunity and Viral Set-Point in Simian Immunodeficiency Virus Disease. Brain Behav. Immun..

[27] Rangassamy M., Athari S.K., Monclus R., Boissier M.-C., Bessis N., Rödel H.G. (2016). Personality Modulates Proportions of CD4+ Regulatory and Effector T Cells in Response to Socially Induced Stress in a Rodent of Wild Origin. Physiol. Behav..

[28] Zozulya A.A., Gabaeva M.V., Sokolov O.Y., Surkina I.D., Kost N.V. (2008). Personality, Coping Style, and Constitutional Neuroimmunology. J. Immunotoxicol..

[29] Kim T.-S., Pae C.-U., Jeong J.-T., Kim S.-D., Chung K.-I., Lee C. (2006). Temperament and Character Dimensions in Patients with Atopic Dermatitis. J. Dermatol..

[30] Gulec M.Y., Gulec H., Oztuna F., Kose S. (2010). Cloninger’s Temperament and Character Dimension of Personality in Patients with Asthma. Int. J. Psychiatry Med..

[31] Heffner K.L., Kiecolt-Glaser J.K., Glaser R., Malarkey W.B., Marshall G.D. (2014). Stress and Anxiety Effects on Positive Skin Test Responses in Young Adults with Allergic Rhinitis. Ann. Allergy. Asthma. Immunol..

[32] Smider N.A., Essex M.J., Kalin N.H., Buss K.A., Klein M.H., Davidson R.J., Goldsmith H.H. (2002). Salivary Cortisol as a Predictor of Socioemotional Adjustment during Kindergarten: A Prospective Study. Child Dev..

[33] Baugh A.T., Senft R.A., Firke M., Lauder A., Schroeder J., Meddle S.L., van Oers K., Hau M. (2017). Risk-Averse Personalities Have a Systemically Potentiated Neuroendocrine Stress Axis: A Multilevel Experiment in Parus Major. Horm. Behav..

[34] Chrousos G.P., Kino T. (2009). Glucocorticoid Signaling in the Cell: Expanding Clinical Implications to Complex Human Behavioral and Somatic Disorders. Ann. N. Y. Acad. Sci..

[35] Miller G.E., Cohen S., Ritchey A.K. (2002). Chronic Psychological Stress and the Regulation of Pro-Inflammatory Cytokines: A Glucocorticoid-Resistance Model. Health Psychol. Off. J. Div. Health Psychol. Am. Psychol. Assoc..

[36] Picard M., Juster R.-P., McEwen B.S. (2014). Mitochondrial Allostatic Load Puts the ‘gluc’ back in Glucocorticoids. Nat. Rev. Endocrinol..

[37] Marketon J.I.W., Glaser R. (2008). Stress Hormones and Immune Function. Cell. Immunol..

[38] Cavigelli S.A., Stine M.M., Kovacsics C., Jefferson A., Diep M.N., Barrett C.E. (2007). Behavioral Inhibition and Glucocorticoid Dynamics in a Rodent Model. Physiol. Behav..

[39] Michael K.C., Bonneau R.H., Bourne R.A., Godbolt L., Caruso M.J., Hohmann C., Cavigelli S.A. (2020). Divergent Immune Responses in Behaviorally-Inhibited vs. Non-Inhibited Male Rats. Physiol. Behav..

[40] Chiurchiù V. (2016). Endocannabinoids and Immunity. Cannabis Cannabinoid Res..

[41] Chiurchiù V., Battistini L., Maccarrone M. (2015). Endocannabinoid Signalling in Innate and Adaptive Immunity. Immunology.

[42] Lu Y., Anderson H.D. (2017). Cannabinoid Signaling in Health and Disease. Can. J. Physiol. Pharmacol..

[43] Maccarrone M., Bab I., Bíró T., Cabral G.A., Dey S.K., Di Marzo V., Konje J.C., Kunos G., Mechoulam R., Pacher P. (2015). Endocannabinoid Signaling at the Periphery: 50 Years after THC. Trends Pharmacol. Sci..

[44] Zhou J., Burkovskiy I., Yang H., Sardinha J., Lehmann C. (2016). CB2 and GPR55 Receptors as Therapeutic Targets for Systemic Immune Dysregulation. Front. Pharmacol..

[45] Boychuk D.G., Goddard G., Mauro G., Orellana M.F. (2015). The Effectiveness of Cannabinoids in the Management of Chronic Nonmalignant Neuropathic Pain: A Systematic Review. J. Oral Facial Pain Headache.

[46] Turcotte C., Chouinard F., Lefebvre J.S., Flamand N. (2015). Regulation of Inflammation by Cannabinoids, the Endocannabinoids 2-arachidonoyl-glycerol and Arachidonoyl-ethanolamide, and Their Metabolites. J. Leukoc. Biol..

[47] Hofmann S.G., Hay A.C. (2018). Rethinking Avoidance: Toward a Balanced Approach to Avoidance in Treating Anxiety Disorders. J. Anxiety Disord..

[48] Loijen A., Vrijsen J.N., Egger J.I.M., Becker E.S., Rinck M. (2020). Biased Approach-Avoidance Tendencies in Psychopathology: A Systematic Review of Their Assessment and Modification. Clin. Psychol. Rev..

[49] Sheynin J., Shind C., Radell M., Ebanks-Williams Y., Gilbertson M.W., Beck K.D., Myers C.E. (2017). Greater Avoidance Behavior in Individuals with Posttraumatic Stress Disorder Symptoms. Stress.

[50] Gellner A.-K., Voelter J., Schmidt U., Beins E.C., Stein V., Philipsen A., Hurlemann R. (2021). Molecular and Neurocircuitry Mechanisms of Social Avoidance. Cell. Mol. Life Sci..

[51] Kaldewaij R., Koch S.B.J., Volman I., Toni I., Roelofs K. (2017). On the Control of Social Approach-Avoidance Behavior: Neural and Endocrine Mechanisms. Curr. Top. Behav. Neurosci..

[52] Toth I., Neumann I.D. (2013). Animal Models of Social Avoidance and Social Fear. Cell Tissue Res..

[53] Kaplan B.L.F. (2013). The Role of CB1 in Immune Modulation by Cannabinoids. Pharmacol. Ther..

[54] Klein T.W., Newton C., Larsen K., Lu L., Perkins I., Nong L., Friedman H. (2003). The Cannabinoid System and Immune Modulation. J. Leukoc. Biol..

[55] Ahn J.J., Abu-Rub M., Miller R.H. (2021). B Cells in Neuroinflammation: New Perspectives and Mechanistic Insights. Cells.

[56] Becher B., Spath S., Goverman J. (2017). Cytokine Networks in Neuroinflammation. Nat. Rev. Immunol..

[57] Parenti A., De Logu F., Geppetti P., Benemei S. (2016). What Is the Evidence for the Role of TRP Channels in Inflammatory and Immune Cells?. Br. J. Pharmacol..

[58] Ramesh G., MacLean A.G., Philipp M.T. (2013). Cytokines and Chemokines at the Crossroads of Neuroinflammation, Neurodegeneration, and Neuropathic Pain. Mediat. Inflamm..

[59] O’sullivan S.E., Kendall D.A. (2010). Cannabinoid Activation of Peroxisome Proliferator-Activated Receptors: Potential for Modulation of Inflammatory Disease. Immunobiology.

[60] Santoni G., Cardinali C., Morelli M.B., Santoni M., Nabissi M., Amantini C. (2015). Danger-and Pathogen-Associated Molecular Patterns Recognition by Pattern-Recognition Receptors and Ion Channels of the Transient Receptor Potential Family Triggers the Inflammasome Activation in Immune Cells and Sensory Neurons. J. Neuroinflamm..

[61] Fowler C.J. (2021). The Endocannabinoid System—Current Implications for Drug Development. J. Intern. Med..

[62] Kasatkina L.A., Rittchen S., Sturm E.M. (2021). Neuroprotective and Immunomodulatory Action of the Endocannabinoid System under Neuroinflammation. Int. J. Mol. Sci..

[63] Struijs S.Y., Lamers F., Rinck M., Roelofs K., Spinhoven P., Penninx B.W.J.H. (2018). The Predictive Value of Approach and Avoidance Tendencies on the Onset and Course of Depression and Anxiety Disorders. Depress. Anxiety.

[64] Tarazona S., Furió-Tarí P., Turrà D., Pietro A.D., Nueda M.J., Ferrer A., Conesa A. (2015). Data Quality Aware Analysis of Differential Expression in RNA-Seq with NOISeq R/Bioc Package. Nucleic Acids Res..

[65] Luo W., Brouwer C. (2013). Pathview: An R/Bioconductor Package for Pathway-Based Data Integration and Visualization. Bioinformatics.

[66] Merico D., Isserlin R., Stueker O., Emili A., Bader G.D. (2010). Enrichment Map: A Network-Based Method for Gene-Set Enrichment Visualization and Interpretation. PLoS ONE.

[67] Yu G., Wang L.-G., Han Y., He Q.-Y. (2012). ClusterProfiler: An R Package for Comparing Biological Themes Among Gene Clusters. OMICS J. Integr. Biol..

[68] R Core Team (2013). R: A Language and Environment for Statistical Computing.

[69] Szklarczyk D., Gable A.L., Lyon D., Junge A., Wyder S., Huerta-Cepas J., Simonovic M., Doncheva N.T., Morris J.H., Bork P. (2019). STRING V11: Protein–Protein Association Networks with Increased Coverage, Supporting Functional Discovery in Genome-Wide Experimental Datasets. Nucleic Acids Res..

